# Checkpoint Inhibitor-Treated Merkel Cell Carcinoma With Cytokeratin 20 (CK20)-Negative Metastatic Immunophenotypic Divergence: Diagnostic and Therapeutic Implications

**DOI:** 10.7759/cureus.115455

**Published:** 2026-08-30

**Authors:** Diego Berlingeri Collazo, Lester Cintron Perez, Emmanuel Torres Troche, Sanjay Vora

**Affiliations:** 1 Internal Medicine, University of Medicine and Health Sciences, Basseterre, KNA; 2 Internal Medicine, McLaren Macomb Hospital, Mount Clemens, USA

**Keywords:** circulating tumor dna, ck20-negative, immune checkpoint inhibitor, immunohistochemistry, immunophenotypic divergence, insm1, merkel cell carcinoma, metastatic neuroendocrine carcinoma, pembrolizumab, tumor heterogeneity

## Abstract

An octogenarian woman with Merkel cell carcinoma of the right upper arm, staged as Stage IIIA at initial diagnosis, underwent wide local excision with sentinel lymph node biopsy followed by adjuvant radiation therapy. She later developed regional nodal recurrence requiring axillary dissection, which was confirmed by biopsy as metastatic Merkel cell carcinoma, requiring axillary dissection. Despite subsequent treatment with pembrolizumab, she developed progressive metastatic disease one year later, with new hepatic and pulmonary lesions.

Biopsy of a hepatic lesion demonstrated a poorly differentiated neuroendocrine carcinoma with a high proliferative index. Notably, immunohistochemistry showed absence of cytokeratin 20 (CK20) expression, raising diagnostic uncertainty regarding lineage confirmation in the setting of CK20-negative metastatic disease. Given the clinical context, the patient was treated with carboplatin and etoposide per established guidelines for high-grade neuroendocrine tumors; however, she exhibited continued disease progression and ultimately transitioned to hospice care, where she died.

This case highlights the diagnostic and therapeutic challenges of advanced Merkel cell carcinoma in the setting of immune checkpoint inhibitor exposure, particularly the potential for loss of the canonical Merkel cell carcinoma immunophenotype at metastatic sites following such therapy. It underscores the importance of integrating clinical history, treatment exposure, and morphologic findings when evaluating atypical metastatic neuroendocrine neoplasms, especially when canonical immunohistochemical markers such as CK20 are absent.

## Introduction

Merkel cell carcinoma is a rare and aggressive primary cutaneous neuroendocrine malignancy arising from mechanoreceptive cells of the dermis. Although it accounts for fewer than 1% of all skin cancers, its incidence has increased substantially over recent decades, and it carries a disease-specific mortality estimated between 33% and 46%, exceeding that of cutaneous melanoma, particularly in advanced or metastatic disease [1,2]. Risk factors include advanced age, immunosuppression, ultraviolet radiation exposure, and integration of Merkel cell polyomavirus, which is detectable in the majority of tumors and confers distinct biological and prognostic implications [1,3].

The histopathological diagnosis of Merkel cell carcinoma relies on a characteristic immunophenotype, most notably cytokeratin 20 (CK20) positivity in a perinuclear dot-like distribution, combined with neuroendocrine marker expression and thyroid transcription factor-1 (TTF-1) negativity [1]. This immunoprofile is critical for distinguishing Merkel cell carcinoma from its most clinically important mimic, metastatic small cell lung carcinoma, which characteristically demonstrates the inverse pattern. CK20 negativity occurs in approximately 5%-10% of confirmed cases and introduces meaningful diagnostic uncertainty, particularly at metastatic sites where the immunophenotype may diverge from the primary tumor [3]. Insulinoma-associated protein 1 (INSM1) has emerged as a highly sensitive nuclear neuroendocrine marker, demonstrating superior sensitivity and interpretability relative to conventional stains in this context [4,5].

The treatment landscape of advanced Merkel cell carcinoma has been transformed by immune checkpoint inhibitors. Pembrolizumab, a programmed cell death protein 1 inhibitor, and avelumab, a programmed death-ligand 1 inhibitor, are both approved and guideline-recommended for first-line management of locally advanced or metastatic disease, with durable objective response rates approaching 50%-56% in pivotal trials [6-9]. Current National Comprehensive Cancer Network (NCCN) guidelines (version 2.2025) designate immune checkpoint inhibitors as preferred first-line therapy, with platinum-etoposide chemotherapy retained as the principal salvage strategy following immunotherapy failure [10]. Despite meaningful advances, acquired and primary resistance to checkpoint blockade remain prevalent, and the mechanisms driving resistance, including transcriptional suppression of human leukocyte antigen (HLA) class I antigen presentation pathways and downregulation of major histocompatibility complex expression, may also be associated with tumor evolution and altered immunophenotypic marker expression, as observed in this case [11,12].

We report a case of advanced Merkel cell carcinoma demonstrating loss of CK20 expression at a metastatic site following immune checkpoint inhibitor therapy, highlighting immunophenotypic evolution while preserving neuroendocrine differentiation. This case raises the clinically important and insufficiently characterized possibility that prolonged immune checkpoint inhibitor exposure may drive immunophenotypic divergence between primary and metastatic disease, with direct implications for pathological diagnosis, circulating tumor DNA (ctDNA) interpretation, and treatment selection in the checkpoint inhibitor-refractory setting.

## Case presentation

An octogenarian woman presented for evaluation of a cutaneous lesion on the posterior aspect of the right upper arm, which had been present for approximately two months and was noted to be progressively increasing in size. She denied pain, bleeding, or systemic symptoms such as fever, weight loss, or fatigue. Her past medical history was significant for chronic obstructive pulmonary disease, hypertension, and a history of multiple cutaneous squamous cell carcinomas of the lower extremities, all of which were treated locally with definitive management. She had a remote history of cigarette smoking, having quit more than 30 years prior, and reported frequent sun exposure. There was no known family history of neuroendocrine malignancy.

A shave biopsy of the lesion demonstrated Merkel cell carcinoma. Histopathologic examination revealed a tumor measuring 0.4 cm with a mixed nodular and infiltrative growth pattern and a thickness of at least 1.3 mm, extending to at least Clark level IV. Ulceration and lymphovascular invasion were not identified. Immunohistochemical staining demonstrated patchy CK20 positivity with a characteristic perinuclear dot-like pattern. Tumor cells were positive for cytokeratin, with weak synaptophysin expression, and negative for TTF-1 and cytokeratin 7, supporting the diagnosis of primary cutaneous Merkel cell carcinoma. Based on the biopsy, the tumor was staged as at least pathological tumor stage 1.

Approximately two months later, the patient underwent wide local excision of the primary lesion with sentinel lymph node biopsy. Pathologic evaluation demonstrated residual Merkel cell carcinoma with a tumor thickness of 1.9 mm and negative surgical margins. An adjacent focus of squamous cell carcinoma in situ was also identified and completely excised. Sentinel lymph node biopsy revealed metastatic Merkel cell carcinoma in one of two lymph nodes examined.

Staging positron emission tomography-computed tomography demonstrated increased metabolic activity in the right axillary region, corresponding to nodular soft tissue measuring approximately 2.1 cm, with a maximum standardized uptake value of approximately 3.0 (Figure 1). This finding was initially interpreted as possibly related to postprocedural changes in the presence of surgical clips; however, nodal disease could not be excluded. Additional findings included a stable, enlarged pre-carinal lymph node measuring 1.2 cm with low-level fluorodeoxyglucose uptake and a 4-mm right lower lobe pulmonary nodule without significant metabolic activity.

**Figure 1:**
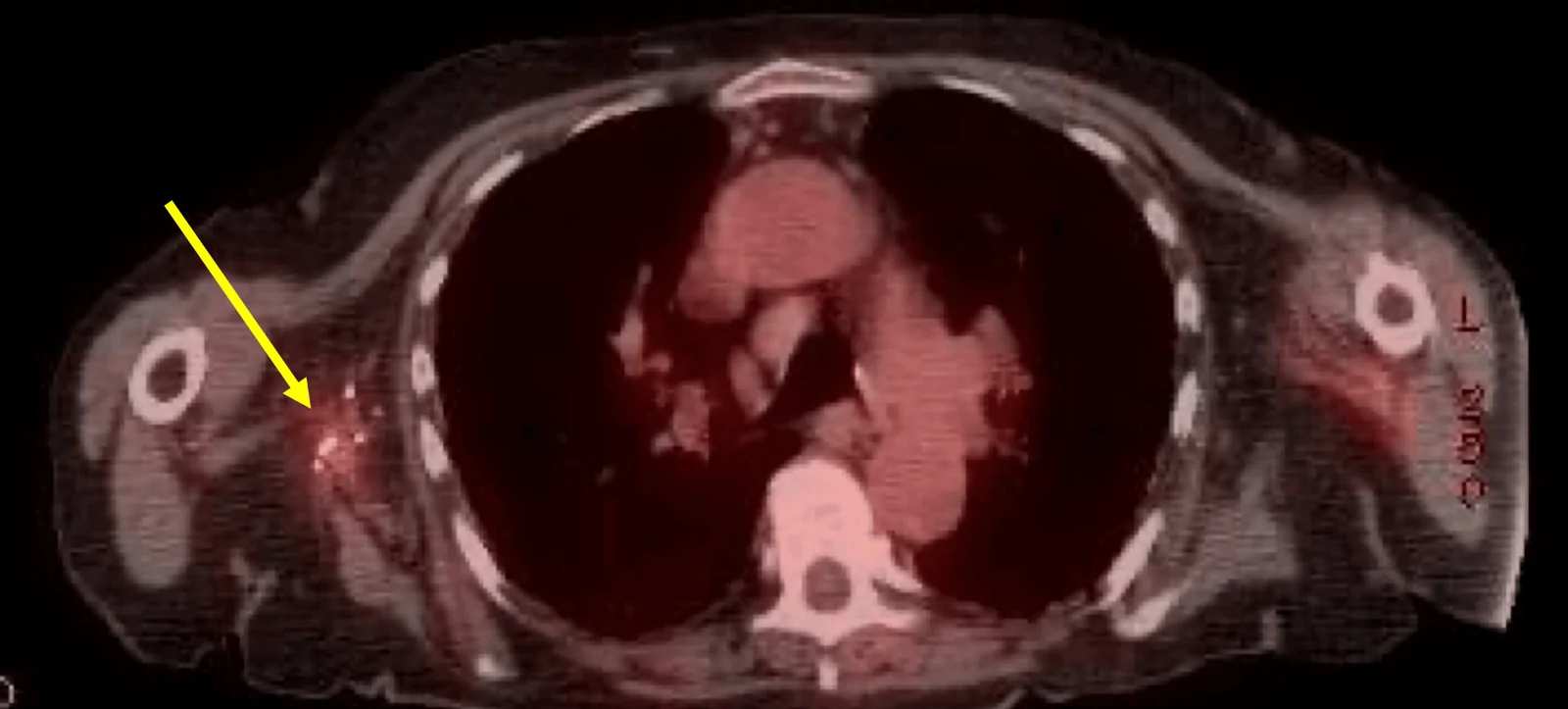
Positron emission tomography-computed tomography demonstrating increased fluorodeoxyglucose uptake within the right axillary region, corresponding to nodular soft tissue adjacent to surgical clips (arrow), initially interpreted as possible postoperative change but concerning for nodal disease

Given nodal involvement, the patient received adjuvant radiation therapy to the primary tumor site and right axillary lymphatic basin, with a total dose of 50 Gy delivered to each region, completed approximately two months after surgical resection.

During surveillance follow-up, ctDNA levels became elevated, prompting further evaluation. Imaging demonstrated findings concerning for recurrent disease within the right axillary lymph nodes. Biopsy confirmed recurrent metastatic Merkel cell carcinoma, and the patient subsequently underwent right axillary lymph node dissection. Pathologic analysis revealed metastatic involvement of nine of 11 lymph nodes, with the largest deposit measuring 2.3 cm. Extranodal extension and extensive lymphovascular invasion were present.

The case was reviewed at a multidisciplinary tumor board, where close surveillance was recommended, with systemic therapy reserved for evidence of distant metastatic disease. The patient subsequently initiated immunotherapy with pembrolizumab at a dose of 200 mg intravenously every three weeks. She completed approximately 13 cycles between April 2024 and July 2025. During treatment, ctDNA levels initially became undetectable. A transient increase occurred when the patient missed two treatment cycles due to hospital operational cancellations, followed by normalization upon resumption of therapy. Serial imaging during this period demonstrated postsurgical changes in the right axilla with mild residual fluorodeoxyglucose uptake but no definitive lymphadenopathy. Additional findings included indeterminate cutaneous nodules of the lower extremities and imaging features consistent with usual interstitial pneumonia, including subpleural fibrosis and honeycombing.

Approximately one year after initiation of pembrolizumab, surveillance imaging demonstrated interval disease progression, with multiple new hypoattenuating hepatic lesions demonstrating increased fluorodeoxyglucose uptake, predominantly within the right hepatic lobe (Figure 2). Additional findings included posterior right lower lobe pleural thickening and nodularity (Figure 3) and a new fluorodeoxyglucose-avid pulmonary nodule in the right upper lobe measuring approximately 8 mm (Figure 4), all concerning for metastatic disease.

**Figure 2:**
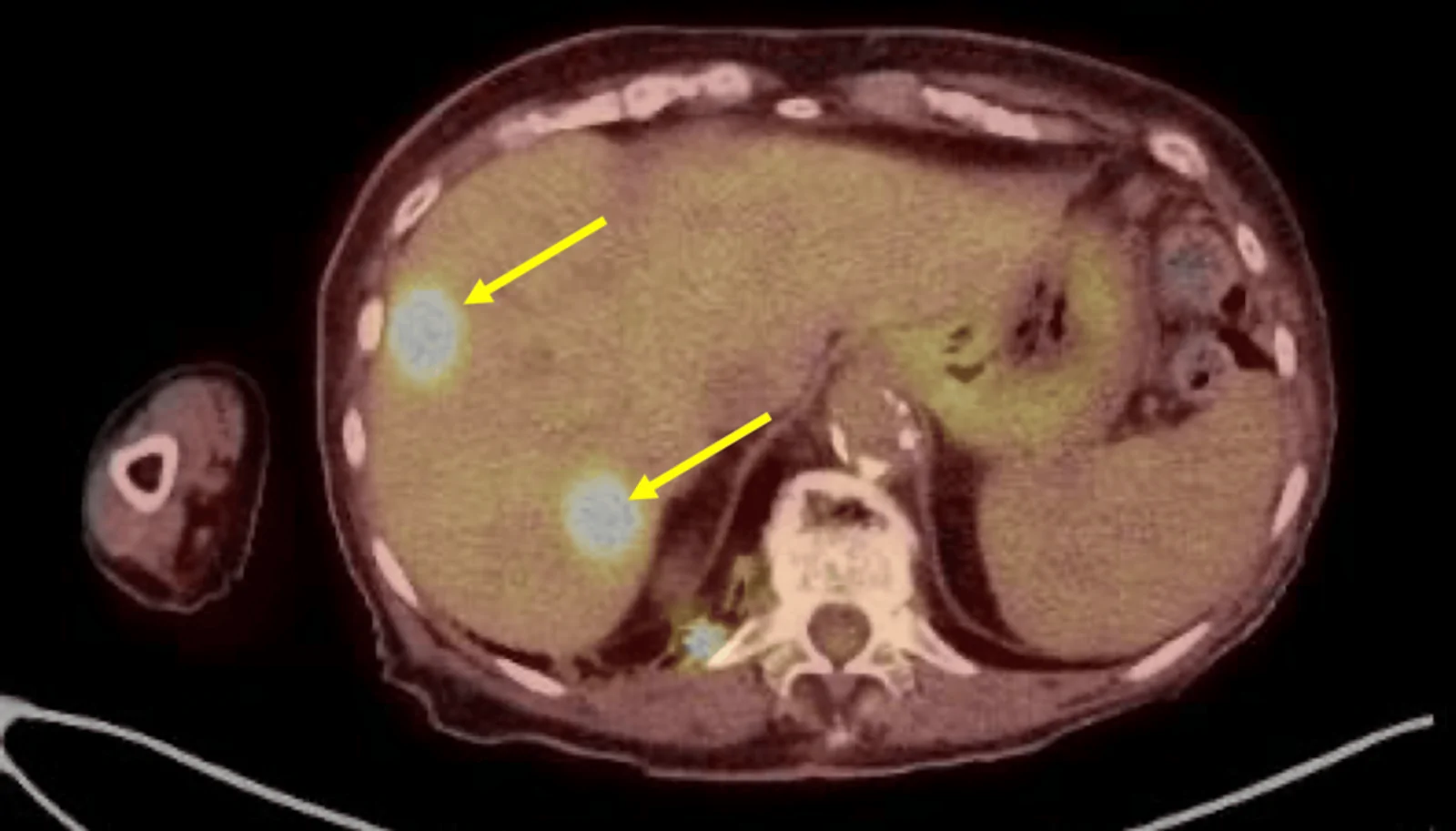
Positron emission tomography-computed tomography demonstrating multiple fluorodeoxyglucose-avid hepatic lesions within the right hepatic lobe (arrows), consistent with metastatic disease

**Figure 3:**
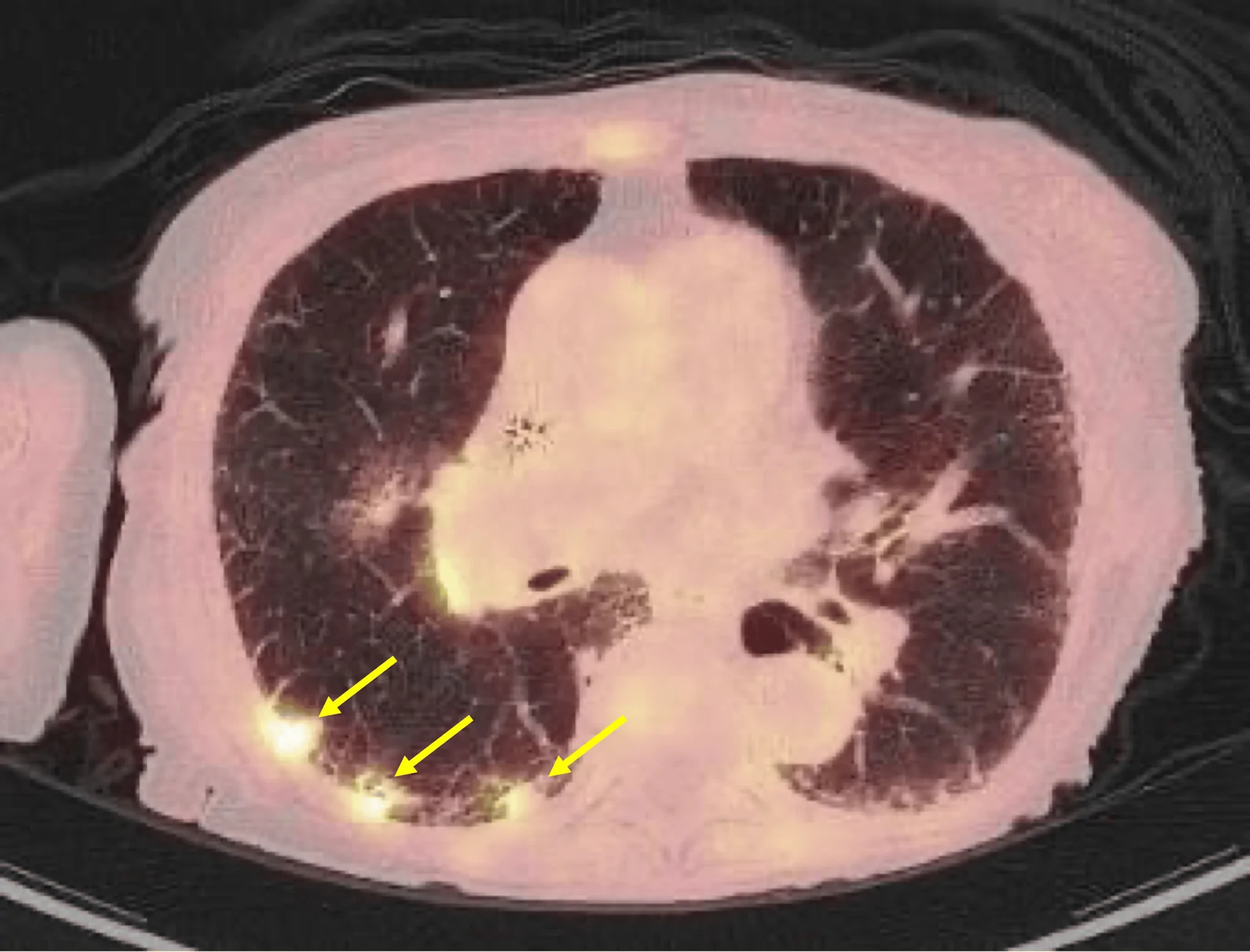
Positron emission tomography-computed tomography demonstrating fluorodeoxyglucose-avid pulmonary nodules and pleural-based lesions within the right lung (arrows), concerning for pulmonary metastatic involvement

**Figure 4:**
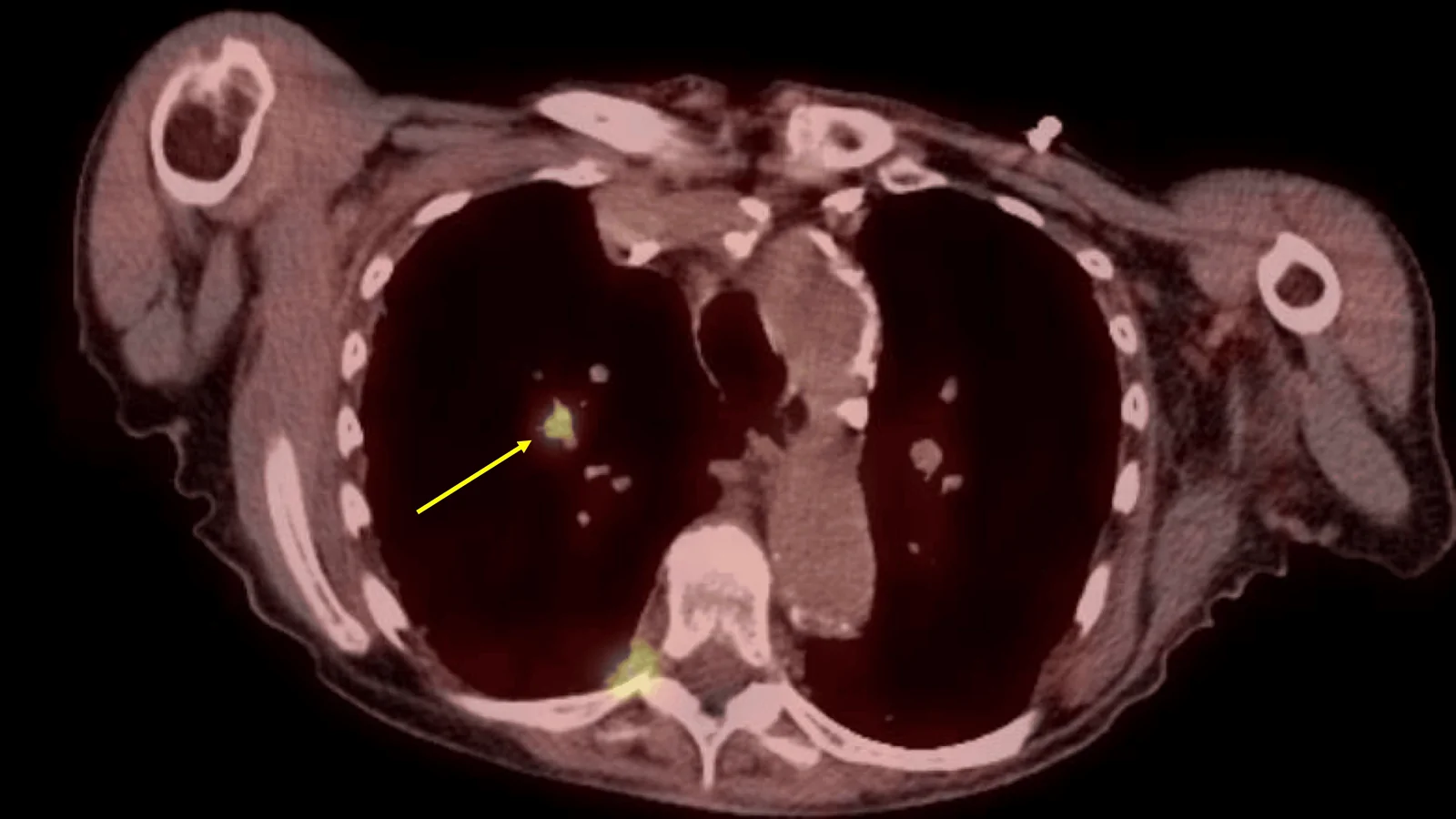
Positron emission tomography-computed tomography demonstrating a fluorodeoxyglucose-avid pulmonary nodule in the right upper lobe measuring approximately 8 mm (arrow), consistent with metastatic disease

To establish histopathologic confirmation, an ultrasound-guided biopsy of a hepatic lesion was performed (Figure 5). The lesion measured 2.32 cm × 1.72 cm (Figure 6). Microscopic evaluation demonstrated liver tissue involved by a poorly differentiated neuroendocrine tumor composed of cells with scant cytoplasm, nuclear molding, focal crush artifact, and increased apoptotic activity, with areas of necrosis. Immunohistochemical staining demonstrated positivity for pancytokeratin (AE1/AE3) and INSM1. Tumor cells were negative for CK20, TTF-1, and synaptophysin. Cluster of differentiation 45 staining was negative in tumor cells and positive in background lymphoid cells. The Ki-67 proliferative index was markedly elevated at approximately 50%-70%, consistent with a highly proliferative malignancy. Compared with the primary tumor, the metastatic lesion demonstrated loss of CK20 and synaptophysin expression while maintaining INSM1 positivity, indicating immunophenotypic divergence rather than loss of neuroendocrine differentiation. The absence of CK20 expression was unexpected given the patient's prior diagnosis of Merkel cell carcinoma and raised diagnostic uncertainty regarding tumor origin.

**Figure 5:**
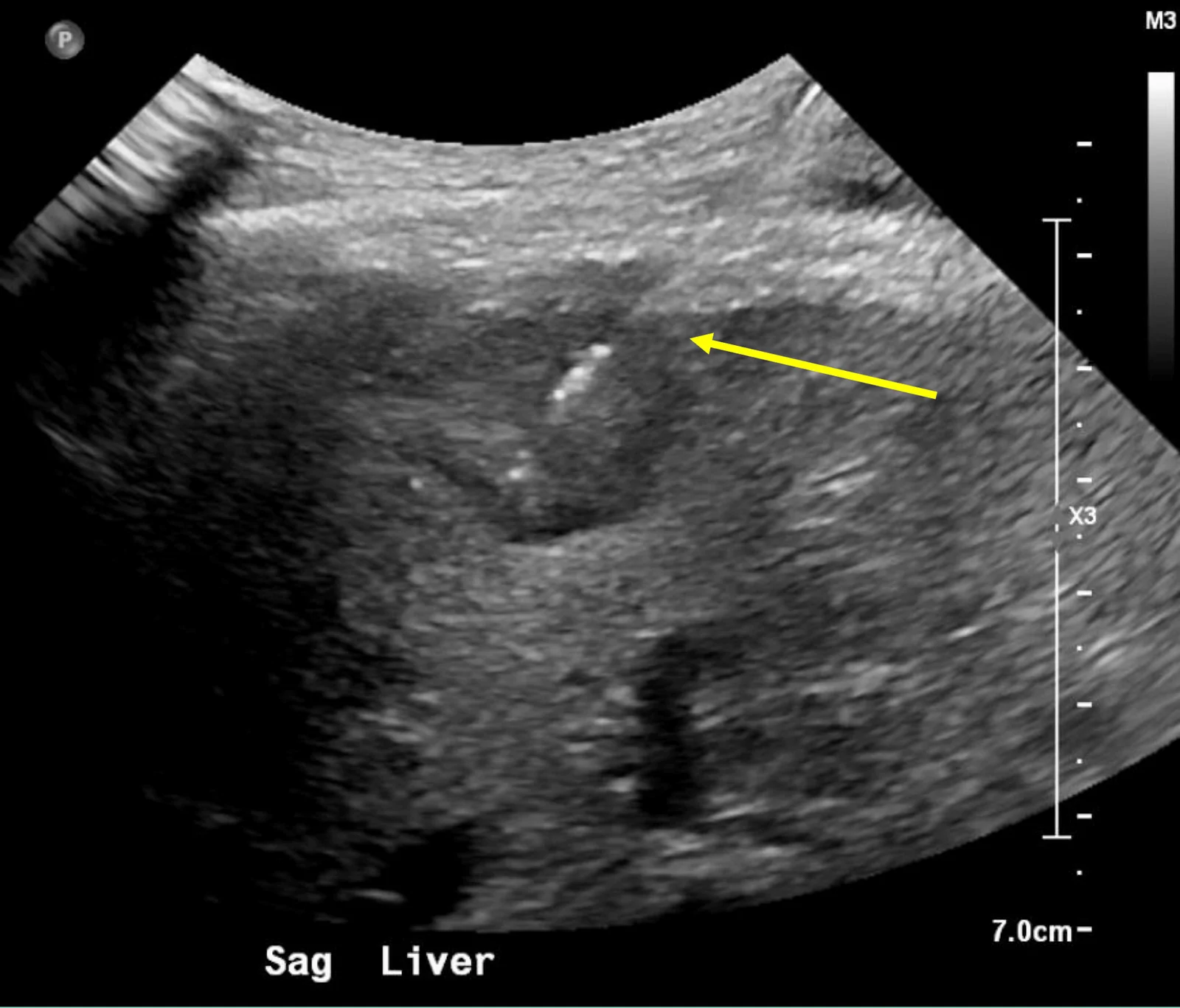
Ultrasound-guided biopsy of a right lobe hepatic lesion demonstrating needle trajectory targeting the hepatic mass suspicious for metastatic disease (arrow)

**Figure 6:**
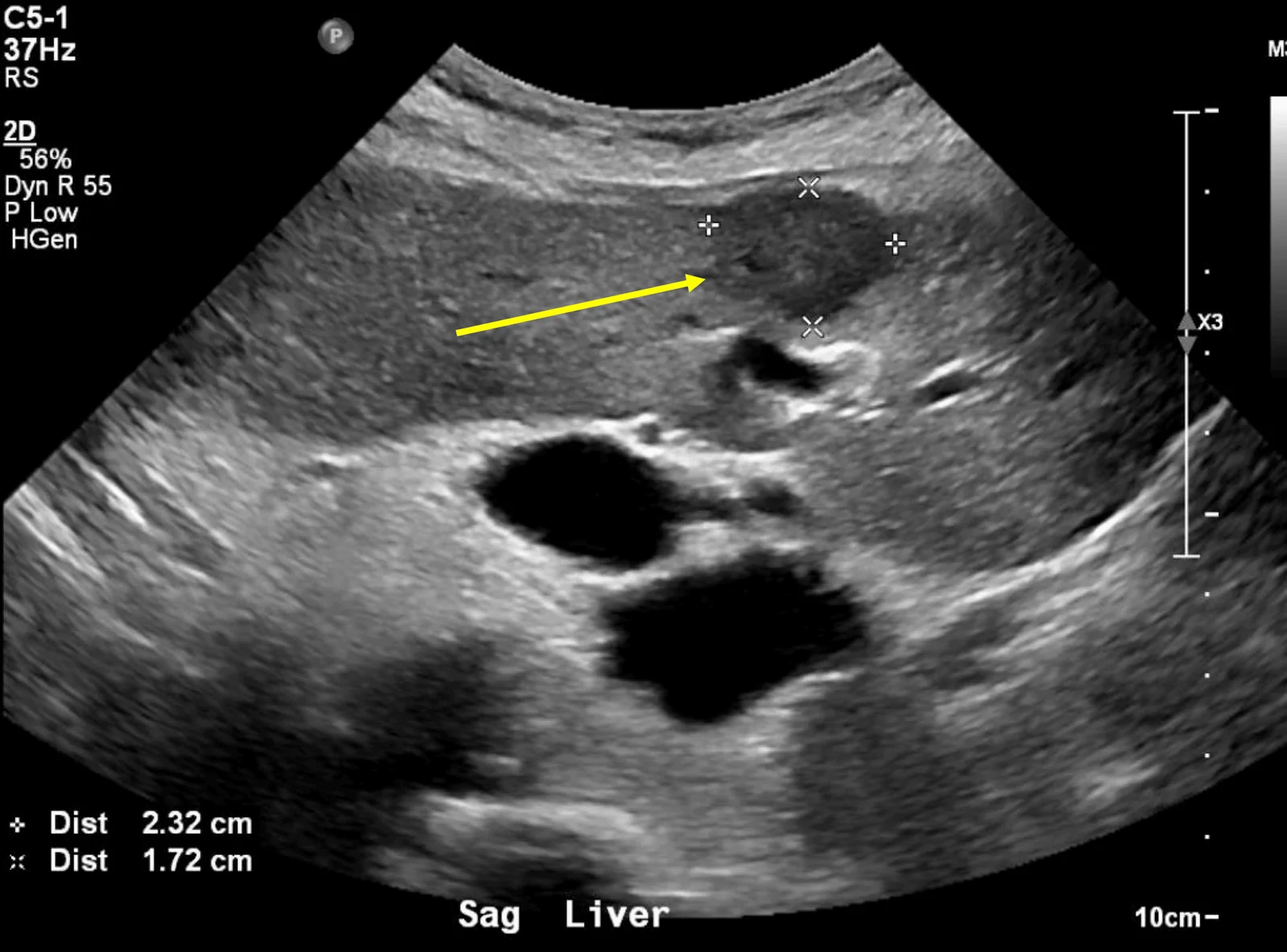
Ultrasound-guided biopsy of a right lobe hepatic lesion showing lesion measuring 2.32 cm × 1.72 cm (arrow)

Given progressive metastatic disease and high-grade neuroendocrine tumor morphology, systemic therapy was transitioned to combination chemotherapy with carboplatin and etoposide. The regimen consisted of carboplatin (area under the curve 5) administered intravenously on day 1 and etoposide 100 mg/m^2^ administered intravenously on days 1-3 of a 21-day cycle. The patient received several cycles between August and December 2025. Follow-up positron emission tomography-computed tomography demonstrated a mixed response, with interval improvement in some right hepatic lesions but progression within the left hepatic lobe (Figure 7), along with persistent pulmonary metastatic disease. Throughout this period, ctDNA remained undetectable despite radiographic progression, raising concern for the emergence of a phenotypically distinct tumor clone not captured by the assay employed.

**Figure 7:**
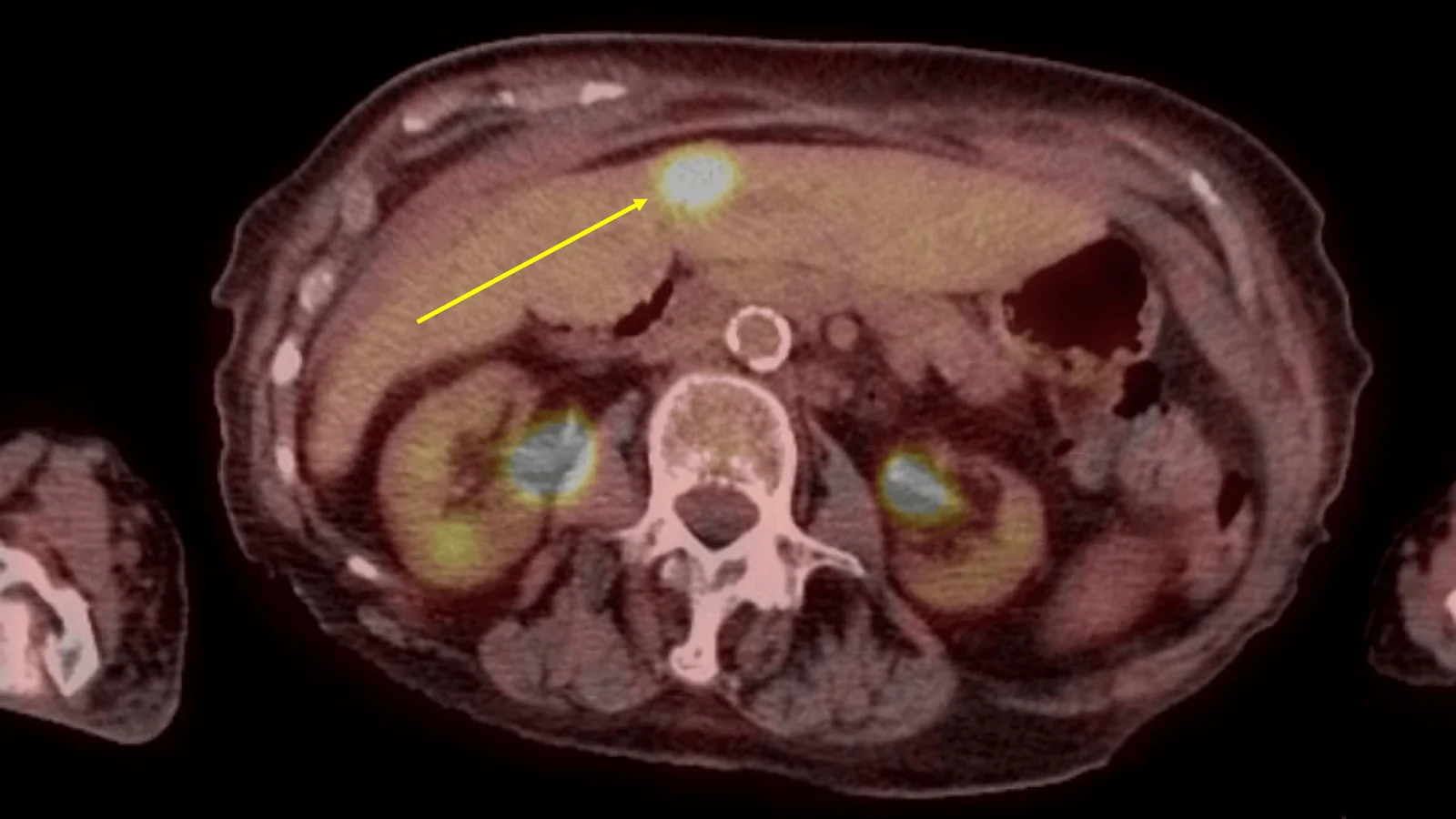
Positron emission tomography-computed tomography demonstrating interval progression of metastatic disease within the liver, with worsening fluorodeoxyglucose-avid lesions in the left hepatic lobe (arrow) compared with prior imaging, consistent with progression of hepatic metastatic involvement despite systemic therapy

Despite ongoing therapy, the patient experienced continued progression of metastatic disease involving the liver and lungs. She developed worsening dyspnea and functional decline and was hospitalized for a symptomatic right-sided pleural effusion requiring repeated ultrasound-guided thoracentesis. Given progressive disease and declining performance status, the decision was made to transition to comfort-focused care. She was managed with palliative measures, including morphine for dyspnea and pain and lorazepam for anxiety. The patient died on March 4, 2026, approximately 33 months after the initial diagnosis of Merkel cell carcinoma, 23 months after the initiation of pembrolizumab therapy, and one week after the transition to hospice. Death was directly attributable to progressive metastatic neuroendocrine carcinoma, clinically consistent with disseminated Merkel cell carcinoma given the overall disease trajectory, prior pathological diagnoses, anatomical distribution of metastases, and response pattern to antineoplastic therapy. A comprehensive clinical timeline of the patient’s diagnostic evaluation, treatment course, and disease progression is presented in Table 1.

**Table 1:** Clinical timeline of the patient’s diagnostic evaluation, treatment course, phenotypic shift, and transition to hospice care MCC, Merkel cell carcinoma; CK20, cytokeratin 20; pT1, pathological tumor stage 1; SCC, squamous cell carcinoma; PET/CT, positron emission tomography-computed tomography; FDG, fluorodeoxyglucose; SUVmax, maximum standardized uptake value; ctDNA, circulating tumor DNA; ALND, axillary lymph node dissection; IV, intravenously; INSM1, insulinoma-associated protein 1; TTF-1, thyroid transcription factor-1; NCCN, National Comprehensive Cancer Network; CT CAP, computed tomography of the chest, abdomen, and pelvis

Time point	Event
~2 months prior to surgery	Shave biopsy: MCC confirmed (0.4 cm, Breslow ≥1.3 mm, Clark level IV; CK20 patchy positive; pT1)
~2 months after diagnosis	Wide local excision + sentinel lymph node biopsy: residual MCC (Breslow 1.9 mm, margins negative); adjacent SCC in situ; one of two sentinel nodes positive
~1 month postsurgery	PET/CT: increased FDG uptake in the right axilla (Figure 1; 2.1 cm, SUVmax ~3.0); stable precarinal node (1.2 cm, SUVmax 2.0); 4-mm right lower lobe nodule
~2 months postsurgery	Adjuvant radiation: 50 Gy to primary site and right axillary basin. Completed ~two months after resection
~8 months after radiation	Elevated ctDNA + PET/CT: right axillary nodal recurrence confirmed. Right ALND: nine of 11 nodes positive, largest 2.3 cm, extranodal extension, extensive lymphovascular invasion
Shortly after ALND	Multidisciplinary tumor board: surveillance recommended; systemic therapy reserved for distant metastatic recurrence
~1 month post-ALND	Pembrolizumab 200 mg IV every three weeks initiated
~6 weeks into treatment	ctDNA undetectable (initial biochemical response)
~5 months into treatment	ctDNA rose transiently (186.6 ME/mL); patient missed two pembrolizumab cycles due to hospital operational cancellations. PET/CT: bilateral inguinal nodes low-level FDG; post-surgical right axilla; no lymphadenopathy
~6 months into treatment	PET/CT: Resolution of right axillary FDG activity; FDG-avid cutaneous nodules bilateral lower extremities (new nodularity right ankle). Suspicious for cutaneous recurrence
~10 months into treatment	ctDNA returned to 0.00 (biochemical remission). Thirteen pembrolizumab cycles completed
~11 months into treatment	PET/CT: new subpleural FDG-avid right lower lobe nodule, possibly inflammatory. CT chest in three months recommended
~15 months into treatment	PET/CT: Disease progression (multiple new FDG-avid hepatic lesions; right lobe predominant; Figure 2); pulmonary nodules and pleural-based lesions within the right lung (Figure 3); new 8-mm right upper lobe nodule (Figure 4)
~16 months into treatment	Liver biopsy: Poorly differentiated neuroendocrine tumor (INSM1+, AE1/3+; CK20-, synaptophysin-, TTF-1-; Ki-67 50%-70%). Carboplatin + etoposide initiated per NCCN guidelines
~2 months after chemotherapy	PET/CT: Mixed response (right hepatic improvement, left hepatic worsening (Figure 7), persistent chest disease; new FDG uptake in true vocal cords)
~4 months after chemotherapy	CT CAP: Interval progression (increased pulmonary metastases, mediastinal/hilar lymphadenopathy, hepatic lesions; new splenic hypodensities; dilated main pulmonary artery)
Final weeks	Hospitalization for dyspnea; two thoracenteses for recurrent right pleural effusions. SCC bilateral lower extremities noted. Transitioned to comfort care and inpatient hospice. Death approximately one week after hospice transition

## Discussion

This case encapsulates several of the most diagnostically and therapeutically challenging dimensions of advanced Merkel cell carcinoma, a rare and aggressive cutaneous neuroendocrine malignancy with significant recurrence risk and unmet therapeutic needs [2]: immunophenotypic evolution with loss of canonical markers at metastatic sites following checkpoint inhibitor therapy, acquired resistance to immune checkpoint inhibition, and the diagnostic uncertainty that arises when canonical histochemical markers are no longer reliable.

The histopathological diagnosis of Merkel cell carcinoma is supported by a characteristic immunoprofile, including CK20 positivity in a perinuclear dot-like pattern, absence of TTF-1, and expression of neuroendocrine markers such as INSM1 [1]. This immunophenotypic fingerprint enables critical differentiation of primary Merkel cell carcinoma from its most clinically consequential diagnostic mimic, metastatic small cell lung carcinoma, which characteristically demonstrates TTF-1 positivity and CK20 negativity. The original cutaneous tumor in the present case conformed to this expected profile, with patchy CK20 positivity in a perinuclear dot pattern, weak synaptophysin expression, and TTF-1 negativity, findings unambiguously supportive of a primary cutaneous Merkel cell carcinoma.

CK20 negativity is encountered in approximately 5%-10% of Merkel cell carcinoma cases and introduces meaningful diagnostic uncertainty, as demonstrated in this patient, where loss of CK20 expression at the metastatic site complicated confirmation of tumor origin [3]. A landmark investigation by Miner et al. established that CK20-negative Merkel cell carcinomas are disproportionately Merkel cell polyomavirus-negative, with 77% of CK20-negative cases lacking detectable polyomavirus, suggesting that CK20 loss may reflect a virally distinct tumor subtype with potentially altered molecular biology [3]. In the metastatic hepatic specimen in the present case, tumor cells were positive for pancytokeratin (AE1/AE3) and INSM1 but negative for CK20, TTF-1, and synaptophysin. The demonstrated absence of synaptophysin expression, compared with its weak expression in the primary tumor, further supports immunophenotypic divergence between primary and metastatic disease. INSM1, a zinc-finger transcription factor expressed during terminal neuroendocrine differentiation, has emerged as a highly sensitive nuclear marker for Merkel cell carcinoma, demonstrating 100% sensitivity across multiple cohorts and superior interpretability relative to synaptophysin and CK20 [4]. Importantly, INSM1 positivity in the metastatic lesion confirms preserved neuroendocrine differentiation despite loss of CK20 expression. Critically, INSM1 cannot distinguish primary cutaneous from metastatic extracutaneous neuroendocrine carcinomas, reinforcing the necessity of clinicopathological integration [4].

Of direct relevance to this case is the documented phenomenon of immunophenotypic drift during disease progression. Shalin et al. reported a case of Merkel cell carcinoma in which the primary tumor demonstrated CK20 positivity but a subsequent brain metastasis exhibited complete loss of CK20 expression with paradoxical acquisition of TTF-1 positivity, a pattern that would conventionally suggest a pulmonary primary and illustrates the degree of immunophenotypic plasticity that Merkel cell carcinoma may exhibit during progression [13]. These cases collectively demonstrate that the canonical Merkel cell carcinoma immunoprofile cannot be assumed to be preserved across metastatic sites or following systemic therapeutic perturbation and that a prior histopathologically confirmed diagnosis should be accorded substantial weight when interpreting atypical neuroendocrine tumor morphology in subsequent biopsies. However, such cases remain rare, and the clinical significance of marker loss at metastatic sites remains incompletely understood.

Pembrolizumab has substantially altered the treatment landscape of advanced Merkel cell carcinoma. In the pivotal KEYNOTE-017 trial, pembrolizumab achieved an objective response rate of 56%, including a 24% complete response rate, in previously untreated advanced disease [6]. Concurrent regulatory approval of avelumab further established immune checkpoint blockade as the therapeutic cornerstone of this disease [7]. Subsequent studies confirmed durable activity in the same setting, with an objective response rate of 49%, a median duration of response of 39.8 months, and an estimated 69% of responders remaining in response at 24 months [9].

The mechanisms underlying acquired immune checkpoint inhibitor resistance in Merkel cell carcinoma are multifaceted and incompletely characterized. Paulson et al. identified dynamic transcriptional suppression of specific HLA class I genes as a mechanism of late relapse, distinguished from irreversible genetic HLA loss by its potential reversibility with pharmacological intervention [11]. Separately, major histocompatibility complex class I downregulation has been documented in 74%-84% of Merkel cell carcinoma tumors at baseline, suggesting that antigen presentation pathway dysregulation is a pervasive and early feature of immune evasion [12].

The present case raises the hypothesis that sustained immune checkpoint inhibition may be associated with the selection of tumor subclones exhibiting altered immunophenotypic marker expression, including downregulation of lineage-defining markers such as CK20, although causality has not been established. Under selective pressure from pembrolizumab-reinvigorated cytotoxic T lymphocytes, subclonal populations harboring diminished CK20 expression or defective antigen-presenting machinery may achieve preferential outgrowth, yielding a CK20-negative metastatic phenotype. Alternatively, checkpoint inhibitor-induced epigenetic reprogramming under sustained immunological pressure may alter cytokeratin expression patterns without requiring clonal selection. The temporal relationship between pembrolizumab exposure and the emergence of a CK20-negative, high-grade neuroendocrine phenotype at metastatic sites is compelling and has not been extensively characterized in the published literature. The transient ctDNA elevation observed following missed pembrolizumab cycles, with subsequent renormalization upon treatment resumption, further illustrates the dynamic interplay between immunological tumor suppression and subclinical tumor kinetics and underscores the importance of treatment continuity in checkpoint inhibitor regimens.

Serial ctDNA monitoring was employed throughout this patient's clinical course as an adjunct to cross-sectional imaging. This approach is supported by a large-scale prospective study demonstrating that ctDNA positivity during surveillance identifies patients at substantially elevated risk of clinical recurrence, with hazard ratios ranging from 6.8 to 20 [14]. A subsequent prospective multicenter study further characterized the temporal relationship between ctDNA positivity and clinical recurrence, reporting a median lead time of 2.7 months from first positive ctDNA to clinically confirmed disease and a hazard ratio of 18.1 for recurrence among ctDNA-positive patients [15]. In this patient, the paradoxical persistence of undetectable ctDNA levels despite radiographically confirmed visceral progression likely reflects the emergence of a phenotypically distinct subclone with altered tumor-shedding biology, highlighting the limitations of ctDNA assays, which may not capture immunophenotypically transformed disease. A persistently undetectable ctDNA level should therefore not preclude continued radiographic surveillance in high-risk patients, particularly in the setting of prior checkpoint inhibitor therapy.

Published reports of immunophenotypic transformation in Merkel cell carcinoma remain limited but collectively demonstrate that CK20 loss at metastatic sites is a recognized, if uncommon, phenomenon with direct diagnostic implications. Shalin et al. described a case in which a CK20-positive primary Merkel cell carcinoma gave rise to a brain metastasis that was completely CK20-negative and had paradoxically acquired TTF-1 expression, a profile that would conventionally indicate a pulmonary primary [13]. Miner et al. characterized a cohort of CK20-negative Merkel cell carcinomas and demonstrated that these tumors are disproportionately Merkel cell polyomavirus-negative, raising the possibility of a biologically distinct subtype with potentially different immunological behavior [3]. Calder et al. described a series of CK20-negative, CK7-positive primary neuroendocrine carcinomas of the skin, further expanding the immunophenotypic spectrum encountered in this setting [5]. The present case adds to this body of literature by documenting CK20 loss at the metastatic hepatic site in a patient who received prolonged pembrolizumab therapy, raising the possibility that tumor evolution under immune checkpoint inhibitor therapy may be associated with changes in immunohistochemical marker expression, including loss of CK20 at metastatic sites, without loss of neuroendocrine differentiation. To the authors' knowledge, this precise combination of prior CK20-positive primary disease, immune checkpoint inhibitor-induced clinical remission, and subsequent emergence of a CK20-negative, INSM1-positive visceral metastasis has not been previously described in the published literature.

Current NCCN guidelines (version 2.2025) designate immune checkpoint inhibitors as preferred first-line systemic therapy for locally advanced or metastatic Merkel cell carcinoma [10]. For patients who progress following immunotherapy, cytotoxic chemotherapy with platinum-etoposide combinations remains the principal available option, a strategy extrapolated from small cell lung carcinoma management given the shared neuroendocrine histomorphology and overlapping pathological features [10,16]. Reported objective response rates to carboplatin or cisplatin plus etoposide range from 30% to 60%, but responses are typically short-lived with a median progression-free survival of approximately three to four months [16]. The mixed therapeutic response observed in this patient is consistent with the heterogeneous and transient responses characteristically seen in the immune checkpoint inhibitor-refractory setting and reflects the limited therapeutic options available at this stage of disease. This case further highlights the indispensable value of multidisciplinary tumor board management at each decision-making juncture in this rare malignancy.

## Conclusions

This case highlights a relatively uncommon presentation of metastatic Merkel cell carcinoma with loss of CK20 expression following immune checkpoint inhibitor therapy, demonstrating clinically significant immunophenotypic divergence between primary and metastatic disease. Such alterations may complicate the pathologic distinction between recurrent Merkel cell carcinoma and other high-grade neuroendocrine malignancies, particularly in the setting of prior therapy. Importantly, preservation of neuroendocrine differentiation despite loss of canonical markers underscores the need for integrated interpretation of morphology, immunohistochemistry, clinical history, and prior pathology. Awareness of this phenomenon is essential to prevent diagnostic misclassification and to guide appropriate management in patients with advanced, treatment-exposed Merkel cell carcinoma.
